# Megaprosthetic Reconstruction for Pathological Proximal Humerus Fractures: Infection Rates, Prevention Strategies, and Functional Outcomes—A Narrative Review

**DOI:** 10.3390/jcm14217672

**Published:** 2025-10-29

**Authors:** Federica Messina, Cesare Meschini, Maria Serena Oliva, Matteo Caredda, Antonio Bove, Giuseppe Rovere, Antonio Ziranu

**Affiliations:** 1 Orthopaedics and Traumatology Department, Università Cattolica del Sacro Cuore, 00168 Rome, Italy; cesare.meschini@gmail.com (C.M.); antonio.bove01@icatt.it (A.B.); antonio.ziranu@policlinicogemelli.it (A.Z.); 2 Department of Orthopedics, Ospedale Isola Tiberina-Gemelli Isola, 00186 Rome, Italy; mariaserena.oliva@gmail.com (M.S.O.); matteocaredda92@gmail.com (M.C.)

**Keywords:** antibacterial coatings, bone tumor reconstruction, endoprosthetic replacement, functional outcomes, limb salvage surgery, megaprosthesis, periprosthetic joint infection, proximal humerus, silver-coated implants, trevira tube

## Abstract

**Background**: Megaprosthetic replacement is widely used following tumour resection but remains challenged by periprosthetic joint infection (PJI) and variable functional outcomes. This narrative review aims to summarise current evidence on infection rates, prevention strategies, and functional outcomes following proximal humerus megaprosthetic reconstruction. We hypothesise that antibacterial coatings and improved soft-tissue techniques reduce infection rates and enhance functional recovery. **Methods**: A comprehensive narrative review of PubMed, Web of Science, and the Cochrane Library was performed using the terms proximal humerus, shoulder, bone tumor, sarcoma, neoplasm, infection, megaprosthesis, and endoprosthetic replacement. Reference lists were screened manually. Case reports and series with fewer than five patients were excluded. Twenty-seven clinical studies (more than 1100 patients; mainly osteosarcoma, chondrosarcoma, and metastatic lesions) were included and qualitatively analyzed. **Results**: The reported infection rates ranged from 4% to 20%, with higher risk in patients receiving adjuvant therapy. Silver-coated implants reduced PJI compared with uncoated designs (e.g., 11.2% → 9.2% in primary implants; 29.2% → 13.7% in revisions) without systemic toxicity. Alternative antibacterial coatings (e.g., silver- or copper-enriched hydroxyapatite) showed promising early results but remain supported by limited clinical data. Soft-tissue stabilization with Trevira tube or synthetic mesh improved joint stability and did not increase infection risk. Functional outcomes, usually assessed by MSTS or TESS, were moderate to good (≈60–80%) overall, with better scores when the deltoid and axillary nerve were preserved or when reverse total shoulder arthroplasty was possible. **Conclusions**: Proximal humerus megaprosthetic reconstruction benefits from meticulous soft-tissue handling, selective use of antibacterial technologies, and multidisciplinary management. The current literature is mainly retrospective, heterogeneous, and non-comparative. Prospective multicenter studies are needed to clarify the long-term effectiveness of silver or alternative coatings, soft-tissue reconstruction techniques, and emerging custom-made 3D-printed prostheses.

## 1. Introduction

The proximal humerus is a common site for primary and metastatic bone tumors, accounting for 7–10% of bone sarcomas. It is the second most frequent site for osseous sarcomas and the third for osteosarcomas. After the femur, the humerus is the second most affected long bone for pathological fractures, with an incidence of 16–27%, significantly impacting load-bearing and daily activities [1,2,3]. Shoulder reconstruction aims to restore stability, preserve deltoid and rotator cuff function when possible, and achieve pain-free mobility to maximize quality of life after major bone loss [4]. Although this review focuses on tumor-related megaprosthetic reconstruction, these broader reconstructive principles provide essential context for understanding the challenges of achieving a stable and functional shoulder.

Surgical treatment for bone tumors largely depends on tumor histology and typically involves wide resection, often resulting in significant bone defects. However, recent advancements in adjuvant therapies, including multi-target chemotherapy and localized radiation, have enabled an increasing number of patients to undergo limb-sparing tumor resections [5,6,7,8].

Several reconstructive options are available, including allografts, alloprosthetic composites (APC), megaprosthetic (megaendoprostetic) replacement (MPR), and reverse shoulder arthroplasty (RSA) [9]. The primary objective of reconstruction is to achieve a stable and functional restoration of the arm and shoulder while minimizing postoperative complications [1,2,3]. Although these principles are shared across different anatomical sites—such as the acetabulum, where surgery aims to relieve pain, restore function, and maintain mechanical stability—the proximal humerus remains uniquely challenging due to its demanding soft-tissue envelope and the critical role of the deltoid and axillary nerve in postoperative function [10].

The recent literature has demonstrated that MPR is the most commonly performed and preferred surgical technique. Given its favorable outcomes, MPR is now considered the gold standard for the treatment of primary bone tumors, pathological fractures, and impending fractures in patients with a good prognosis [1,2,11,12,13,14].

Complications of this surgical approach include soft tissue failure (painful instability, subluxations, dislocations), aseptic loosening, and infection. In the proximal humerus, while subluxations and dislocations are the most common failure causes, infections and mechanical complications are also significant, though less frequent [1,13,15,16,17].

This narrative review aims to summarise current evidence on infection rates, prevention strategies, and functional outcomes following proximal humerus megaprosthetic reconstruction. We hypothesize that antibacterial coatings and improved soft-tissue techniques reduce infection rates and enhance functional recovery.

## 2. Materials and Methods

A comprehensive literature search was performed in PubMed, Web of Science, and the Cochrane Library to identify studies addressing causes of failure in proximal humeral megaprostheses. The following keywords and their combinations were used in an exploratory way: *proximal humerus*, *shoulder*, *bone tumor*, *sarcoma*, *neoplasm*, *infection*, *megaprosthesis*, *endoprosthetic replacement*. Additional relevant articles were identified by manually screening the reference lists of included studies. No restrictions were applied regarding publication year or study design.

Studies were considered if they were published in English and reported proximal humerus reconstruction with megaprostheses, including data on infection or failure modes and, when available, functional outcomes. Case reports with fewer than five patients, studies lacking specific data on the proximal humerus, and articles without original clinical data were excluded.

Two authors (Messina F., Meschini C.) independently screened titles, abstracts, and full texts; disagreements were resolved by consensus or, when necessary, with a senior author (Ziranu A.). Journal titles, author names, and institutional affiliations were not concealed at any stage, and no direct contact was made with study authors for patient-specific data.

**Figure 1:**
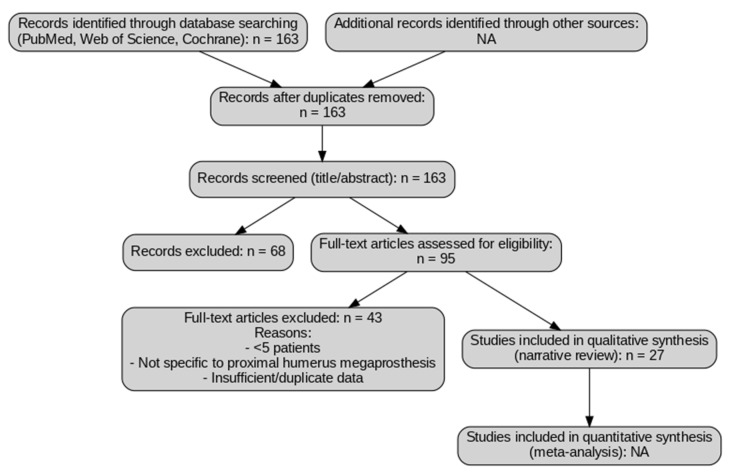
Study selection flow diagram for narrative review. The diagram illustrates the selection process of the studies included in this review. A total of 163 records were identified through database searching (PubMed, Web of Science, and Cochrane Library). After screening titles and abstracts, 95 full-text articles were selected, of which 43 were excluded for the following reasons: small sample size (<5 patients), lack of specific focus on proximal humerus megaprosthesis, or insufficient/duplicate data. Finally, 27 studies were included in the qualitative synthesis. No quantitative synthesis (meta-analysis) was performed, as this work is a narrative review. NA: Not applicable.

Because this work is a narrative review and not a formal systematic review, the search and selection process was designed to be broad and inclusive but not protocol-driven. A simplified flow diagram (Figure 1) is provided for transparency to illustrate how records were screened and excluded; however, this does not represent a PRISMA-guided systematic review, and no quantitative synthesis or formal risk-of-bias assessment were performed (Figure 1).

### Use of Generative AI

Generative artificial intelligence tools (ChatGPT, OpenAI) were used to improve the English language and clarity of the text. All content was reviewed and approved by the authors, who take full responsibility for the final manuscript.

## 3. Results

A total of 163 articles were initially identified through the literature search strategy. After removing duplicates and screening titles and abstracts, 95 studies were selected for full-text review. Of these, about 25–30 studies were included in this narrative synthesis. Several studies were excluded at this stage due to small sample sizes (<5 patients), lack of data specific to proximal humerus reconstruction, absence of a focus on megaprosthetic techniques, or insufficient reporting of infection and functional outcomes. The selected literature was then analyzed to provide a cross-sectional overview of the current evidence, emphasizing infection rates, prosthetic innovations, and postoperative functional results in patients undergoing proximal humeral reconstruction. Infection rates ranged from 4% to 20%, with the highest incidence in primary sarcoma patients receiving adjuvant therapy [8,11,16,18]. Functional outcomes were generally moderate to good (MSTS/TESS 60–80%), reaching up to 96% when deltoid and axillary nerve preservation was achieved [19,20,21,22].

Infection rates in proximal humerus megaprostheses varied widely, with higher risk observed in patients with primary bone sarcomas receiving chemotherapy and/or radiotherapy [8,11,16,18,19]. Silver-coated implants consistently demonstrated lower infection rates, particularly in revision settings, without reports of systemic toxicity [20,23,24,25]. Alternative antibacterial coatings such as silver- or copper-enriched hydroxyapatite have shown promising early results but remain based on limited clinical evidence [23,26,27,28].

Functional outcomes, typically assessed using MSTS or TESS scores, were moderate to good (≈60–80%) overall but showed high variability depending on soft tissue preservation and reconstruction type [19,20,21,22]. Emerging approaches such as 3D-printed custom-made prostheses show encouraging mid-term outcomes with improved anatomical fit [23]. These findings underscore the multifactorial nature of achieving durable, infection-free and functionally satisfactory outcomes after proximal humeral tumor resection and megaprosthetic reconstruction, and highlight the need for continued innovation and high-quality clinical studies. 

A detailed summary of infection rates, prosthetic innovations, and functional results is provided in Table 1.

## 4. Discussion

Periprosthetic joint infection (PJI) is a well-documented complication in primary prosthetic implants, occurring in approximately 1–2% of cases. However, when modular endoprostheses (MPR) are used following extensive bone resections in oncologic or revision surgery, the infection rate can rise significantly, reaching up to 50% [25,30,31].

In the context of proximal humerus replacement, PJI appears to be a rare but serious complication, possibly owing to the rich vascular supply and soft-tissue coverage around the shoulder. Henderson et al. reported an overall infection rate of 6.3% in patients with shoulder replacements [32]. Kumar et al. documented five infections (four superficial and one deep) in a cohort of 100 patients, all caused by Staphylococcus aureus [33]. Similarly, Arhens et al. identified one wound dehiscence and three deep infections requiring surgical intervention among 102 patients who underwent shoulder MPR [16]. Cannon et al., in their study on the functional outcomes of 83 patients undergoing total proximal humerus replacement, reported two infections, including one leading to implant removal [34]. A systematic review and meta-analysis by Fiore et al., which included 382 patients, identified nine infections following proximal humeral MPR [13], whereas Ross et al. found no deep infections in their 25-patient series [35].

It is important to note that the available evidence on proximal humerus megaprosthetic reconstruction is highly heterogeneous. Most included studies are retrospective case series with small sample sizes and variable follow-up durations [13,16,20,24]. Moreover, standardized definitions of complications and consistent reporting of functional outcomes (e.g., MSTS, TESS) are often lacking [21,34]. Such variability limits direct comparability and may lead to over- or underestimation of infection rates and functional results. The predominance of non-comparative studies and the absence of robust prospective data further reduce the strength and generalizability of current conclusions [11,13]. Functional outcomes following proximal humeral megaprosthesis are influenced by multiple patient- and procedure-related factors. Extensive resections that sacrifice the deltoid muscle or axillary nerve markedly reduce shoulder stability and motion, whereas preservation of these structures is associated with better function [20,21,36,37]. Recent registry analyses and large multicenter series have provided more robust outcome data for shoulder megaprostheses, particularly reverse configurations, reporting satisfactory mid-term function and manageable complication rates [36,37]. These data support the current trend toward reverse total shoulder arthroplasty when feasible and confirm its ability to achieve good functional outcomes in oncologic reconstruction.

Patient comorbidities and general health status can impair recovery, while timely and structured postoperative rehabilitation plays a critical role in restoring mobility [34]. Furthermore, procedures performed in high-volume oncologic centers by experienced surgical teams have been linked to improved functional scores, highlighting the impact of surgical expertise and multidisciplinary care on postoperative outcomes [13,16].

To mitigate PJI incidence and improve treatment outcomes, several strategies have been developed. One of the most explored approaches involves modifying implant surfaces with antibiotics, antiseptics, and metal coatings, aiming to reduce bacterial colonization and enhance infection resistance [25,38].

### 4.1. Overall Summary and New Insights

This Narrative Review provides a comprehensive synthesis of current evidence regarding infection rates, preventive strategies, and functional outcomes after proximal humerus megaprosthetic reconstruction. Reported infection rates ranged from 4% to 20%, with the highest risk in patients receiving adjuvant therapies for primary sarcomas [8,11,16,18,19]. Silver-coated implants and other antibacterial surface modifications consistently demonstrated reduced periprosthetic infection rates, particularly in high-risk or revision cases, while maintaining safety [20,21,22,24,30,39]. Soft-tissue reconstruction techniques—especially the use of the Trevira tube and synthetic meshes—proved effective in enhancing joint stability without increasing infection risk [22,40,41,42,43]. Functional outcomes were generally moderate to good, reaching up to 96% in reverse configurations with preserved deltoid and axillary nerve function [19,21,28,29,36,37].

Compared with previous research, this review adds a focused, anatomy-specific perspective that integrates both infection control and functional recovery strategies in a single framework. It highlights the importance of multidisciplinary surgical management and the emerging role of antibacterial coatings and 3D-printed custom designs in improving oncological limb-salvage outcomes [23,25,26,27,44,45,46].

### 4.2. Silver Coat

Several in vitro and animal studies have demonstrated the efficacy and safety of silver in preventing periprosthetic infections [26]. In orthopedic prostheses, three commercially available silver-coated modular endoprostheses (MPRs) are designed to reduce infection risk through different coating technologies [26]:

- MUTARS^®^ MPR (Implancast, Buxtehude, Germany) contains the highest silver content among available prostheses. It consists of a titanium-vanadium (TiAl6V4) base, coated with two metallic layers: an inner 0.2 mm gold layer, which facilitates the controlled release of silver ions into periprosthetic tissues, and an outer 15 μm pure silver layer applied via galvanic deposition [24,26].
- METS^®^ prosthesis (Stanmore Implants, Elstree, UK) is originally made of titanium and features a 5 μm silver layer, known as Agluna^®^ (Accentus Medical, Oxfordshire, UK), created through anodization and silver absorption via ion exchange [5].
- Megasystem C^®^ MPR (Waldemar Link, Hamburg, Germany) incorporates PorAg^®^ (Porous Argentum, New York, NY, USA) technology, consisting of a 1 μm deep layer containing silver and an outer 0.1 μm TiAg20N layer. These layers generate an electrochemical reaction, forming an electron cloud around the prosthetic surface. The resulting cathodic reaction disrupts ATP synthesis in bacteria, leading to cell death [30,47].

Clinical studies have evaluated the effectiveness of silver-coated implants in preventing infections following proximal humerus replacement, with promising results.

No infections were reported in Trovarelli et al.’s case series of 22 patients or Schmolders et al.’s study of 30 patients using silver-coated implants [20,25]. Similarly, Perry et al., in a single-center study of 50 patients, observed only one infection, which occurred in the non-coated prosthesis group [20,25].

Beyond reducing early periprosthetic infections, silver-coated implants have been associated with less invasive infection management, a lower likelihood of implant removal, and a reduced risk of amputation. Given these benefits, their most promising use appears to be in revision surgery for infection-related implant failures, where effective infection control is crucial [39] (Table 2).

#### Other Antibacterial Coating Strategies

In addition to silver coatings, alternative antibacterial strategies for megaprosthetic implants have gained attention. Among these, hydroxyapatite coatings enriched with copper (Cu-HA) have demonstrated promising antimicrobial effects while preserving osteoconductivity, offering a potential alternative in patients where silver-related side effects are a concern [28]. Similarly, silver-containing hydroxyapatite composites (Ag-HA) continue to be explored, particularly for their dual action in infection prevention and bone integration, showing encouraging outcomes in various orthopaedic settings [27]. Furthermore, emerging nanostructured coatings—including antibiotic-loaded hydrogels and self-assembling surfaces—are being investigated to provide a controlled local release of antimicrobial agents and to disrupt early biofilm formation [29,44,45,46]. Despite their promise, these technologies remain supported by low-level and mid-term evidence, and their widespread adoption is currently limited by high production costs, regulatory hurdles, and the lack of long-term clinical data. Future prospective multicenter studies are needed before these coatings can be routinely recommended in oncologic megaprosthese.

### 4.3. Trevira Tube

Reconstructing the upper limb with modular tumor endoprostheses is a well-established approach for managing primary malignant bone tumors or metastatic disease. However, functional outcomes following upper limb surgery with megaendoprostheses are often less than ideal. This is largely due to the frequent need for axillary nerve resection, resulting in the loss of deltoid muscle function, as well as the common involvement of the rotator cuff [48].

To address joint instability in such cases, the Trevira tube, a knitted polyester structure, has been developed to facilitate the reattachment of remaining muscles and tendons. This allows for fibroblastic ingrowth, contributing to the development of a stable joint [40].

Despite its functional advantages, concerns have been raised regarding the risk of deep tissue infections associated with polyester fibers. However, the current literature provides limited data on soft tissue reconstruction using a Trevira tube following proximal humerus replacement. Schmolder et al. investigated the role of the Trevira tube in 30 patients undergoing endoprosthetic replacement of the proximal humerus after wide resection [25]. Among them, 15 patients received an endoprosthesis combined with a Trevira tube, while in the remaining 15, the surgeon opted against using the tube due to an already stable joint. They reported only one case of periprosthetic joint infection (PJI), occurring after implant revision for luxation in the subgroup with a Trevira tube. However, the Trevira tube was not associated with a statistically significant increase in periprosthetic infection rates [25].

Similarly, Gosheger et al. analyzed 69 cases of megaprosthesis implantation with use of a Trevira tube for soft tissue reattachment around the megaprosthesis, including 16 proximal humerus replacements. Their findings indicated no increased infection rate compared to replacements without the Trevira tube. Additionally, the Trevira tube effectively prevented dislocation [40]. A recent review has also emphasized the critical role of soft-tissue reattachment and biointegration strategies—such as synthetic meshes and polyester sleeves—in enhancing joint stability and potentially reducing infection risk after megaprosthetic reconstruction [41].

In another study, three patients underwent proximal and/or total humerus replacement using the MUTARS system with Trevira tube capsular reconstruction. The Trevira tube successfully prevented dislocation without increasing the infection rate [42].

Henderson et al. reported a 6.3% infection rate in patients with proximal humerus replacements, which was lower than that observed in some series [32]. A higher complication rate was noted in more physically active patients. Tang et al. found that synthetic mesh-based soft tissue reconstruction resulted in lower complication rates and better functional outcomes. Similar observations were made in the current study, where initial soft tissue reconstruction with synthetic mesh was associated with fewer soft tissue complications and infections [43]. Henderson et al. also highlighted that instability-related complications (Type I) are more frequent in patients undergoing shoulder or proximal femur replacement, emphasizing the potential benefits of synthetic mesh in such cases [21] (Table 3).

### 4.4. Tumor Type and Adjuvant Treatment

The risk of periprosthetic joint infection (PJI) in proximal humerus and shoulder megaprostheses is significantly influenced by both the underlying tumor type and adjuvant therapies [17]. Primary bone tumors, such as osteosarcoma and chondrosarcoma, are associated with a higher incidence of PJI compared with secondary metastatic lesions. This increased risk is likely multifactorial: chemotherapy and radiotherapy can impair local vascularity, delay wound healing, and suppress the immune response, while patients with aggressive primary tumors often experience greater soft-tissue compromise and systemic immunosuppression [5,6,11,16,20,24,41,49,50]. These factors together may create a more favorable environment for bacterial colonization and infection development. This increased susceptibility is likely due to the need for extensive resection and soft tissue loss, which compromises the local immune response and delays wound healing [6,50]. In a study analyzing mega-arthroplasty in different joints, patients with primary bone tumors exhibited a statistically significant higher risk of PJI than those with metastatic bone disease, suggesting that the aggressive biological behavior of these tumors contributes to infection susceptibility [5,31].

Adjuvant therapies, including chemotherapy and radiotherapy, further exacerbate the risk of infection. Chemotherapy-induced immunosuppression, leukopenia, and impaired wound healing are well-documented risk factors for surgical site infections (SSI) [6,7]. Moreover, postoperative chemotherapy within one month of surgery has been associated with an increased risk of PJI. Similarly, radiation therapy, while effective in local tumor control, can cause significant soft tissue damage, reduce vascularization, and impair host defenses, thereby increasing infection susceptibility [8].

In a study evaluating the functional and oncological outcomes of inverse proximal humerus prostheses, infections were predominantly observed in patients who had received radiotherapy, reinforcing the notion that radiation-induced tissue damage is a key predisposing factor [5,6,22].

However, the inherent complexity of this patient cohort, coupled with the need for aggressive oncologic therapies and the resultant immunosuppression, continues to pose substantial challenges to effective infection prevention. Ongoing research is essential to refine strategies for infection control, especially in high-risk patients undergoing megaprosthetic reconstruction after tumor excision.

### 4.5. Other Relevant Factors

The recent literature highlights additional factors that may influence outcomes in megaprosthetic reconstructions beyond infection-specific strategies. Theil et al. showed that while longer primary surgeries were linked to mechanical failures, no clear association emerged between surgical duration and infection risk; notably, more prolonged revision surgeries might reduce subsequent failures, possibly due to more extensive debridement efforts [31,51]. Berger et al. reported a 30% infection rate after mega-prosthesis implantation, with persistent infections or amputations occurring in nearly half of infected patients, emphasizing the high morbidity linked to periprosthetic infections in these complex reconstructions [18]. Meanwhile, advanced approaches such as three-dimension-printed custom-made prostheses have demonstrated encouraging mid-term outcomes, potentially improving anatomical fit and reducing mechanical complications [23]. Although blood loss management protocols like tranexamic acid administration have shown benefits in joint arthroplasty [19], their role in tumor-related megaprostheses remains to be fully elucidated. These insights underline the multifactorial nature of complications and the need for tailored, multidisciplinary strategies.

## 5. Conclusions

The current literature on megaprosthetic reconstruction of the proximal humerus is predominantly composed of small, retrospective series with heterogeneous patient populations and variable follow-up durations. As a result, the true incidence of periprosthetic joint infection (PJI) and the long-term functional outcomes remain incompletely defined.

Silver-coated endoprostheses and alternative antibacterial surface treatments show encouraging early results in lowering infection risk, particularly in revision or high-risk settings, but the supporting evidence is limited and largely non-comparative. Similarly, soft-tissue reconstruction techniques such as the Trevira tube appear to improve joint stability without clearly increasing infection rates, although data are still scarce.

Given these limitations, conclusions regarding the superiority of any specific implant design, coating technology, or reconstruction strategy should be drawn cautiously. Well-designed, prospective, multicenter studies with standardized outcome reporting are urgently needed to clarify the long-term effectiveness of antibacterial coatings, soft-tissue reconstruction methods, and modern implant innovations such as 3D-printed custom-made prostheses.

Until such data become available, the management of proximal humeral reconstructions after tumor resection should rely on a multidisciplinary approach, careful patient selection, meticulous soft tissue handling, and judicious integration of emerging technologies to mitigate infection risk and optimize functional outcomes.

## Figures and Tables

**Table 1:** Summary of Key Findings on Infection Rates, Functional Outcomes, and Mechanical Complications in Proximal Humerus Megaprosthetic Reconstruction.

*Outcome*	*Findings*	*References*
***Infection rates***	Ranged from 4% to 20%; higher in patients with primary sarcomas undergoing chemotherapy/radiotherapy.	[8,10,15,18,19]
***Effect of silver-coated implants***	Lower infection rates compared to non-coated implants; reduction from 11.2% → 9.2% (primary) and from 29.2% → 13.7% (revisions). No systemic toxicity or argyria.	[20,24,25,26]
***Alternative coatings***	Silver-hydroxyapatite and copper-enriched hydroxyapatite showed promising antimicrobial effects (experimental/early clinical).	[27,28,29]
***Functional outcomes (MSTS/TESS)***	Generally 61–80%; up to 96% in reverse total shoulder arthroplasty with preserved deltoid and axillary nerve.	[19,20,21]
***3D-printed custom prostheses***	Encouraging mid-term results; improved anatomical fit and lower mechanical complication rates despite stress shielding.	[23]
***Mechanical complications***	Aseptic loosening and periprosthetic fracture are relatively infrequent as revision causes.	[11,27,28]

Abbreviations: MSTS = Musculoskeletal Tumor Society score; TESS = Toronto Extremity Salvage Score.

**Table 2:** Clinical evidence on silver-coated megaprostheses.

*Study*	*Population/Site*	*Design*	*Follow-Up (Months)*	*Main Findings*
***Gosheger 2004*** [26]	Rabbit, femur megaprosthesis (silver coated vs. titanium)	Preclinical animal study	3	PJI 7% silver vs. 47% titanium; no systemic toxicity reported
***Hardes 2010*** [24]	Bone sarcoma, lower limb MPR (silver-coated vs. uncoated)	Retrospective comparative series	60	Lower PJI in silver group; fewer aggressive salvage procedures.
***Trovarelli 2019*** [20]	22 patients with modular reverse total shoulder prosthesis after tumor resection (innervated deltoid preserved)	Retrospective case series	46	No infections reported.
***Schmolders 2017*** [25]	30 patients with proximal humeral endoprosthesis; 15 with Trevira tube vs. 15 without	Retrospective comparative series	36	Very low PJI; Trevira safe with silver.
***Wafa 2015*** [5]	High-risk patients receiving silver-treated endoprostheses (Agluna^®^), mixed anatomical sites	Case-control study	Early postoperative period	Lower early PJI with Agluna^®^ silver vs. control.
***Fiore 2021*** [13]	Meta-analysis, 382 shoulder MPR after oncologic resection	Systematic review and meta-analysis	18–203	PJI ~2–3%; use of synthetic mesh not associated with higher infection risk.
***Ruggieri 2019*** [39]	Review, oncologic reconstructions	Narrative review	—	Silver technology reduces early PJI; particularly useful in revision settings.

Abbreviations: PJI = periprosthetic joint infection; MPR: megaendoprosthetic replacement.

**Table 3:** Clinical results of Trevira tube use in proximal humerus megaprosthesis reconstruction.

*Author (Ref.)*	*Year*	*Design*	*Population*	*Main Findings (FU, Complications, Functional Outcomes)*
***Gosheger et al.*** [40]	2001	Retrospective case series	69 megaprostheses (16 PH) with Trevira tube for capsular/muscle reattachment	FU: 31.6 mo (9–78). Complications: 0/16 dislocation (PH); 6/69 inf. (8.7%); 1/69 AL Functional outcome: MSTS in PH 70.4% (46–83%)
***Gosheger et al.*** [42]	2005	Retrospective case series	3 patients (PH/TH) MUTARS with Trevira tube + trapezius/latissimus transfer	FU: 12–18 mo. Complications: No wound compl.; stable articulation. Functional outcome: ROM similar to controls: Abd 35–40°, Flex 30–40°
***Schmolders et al.*** [25]	2017	Retrospective comparative series	30 patients (15 Trevira, 15 no Trevira) 30 patients PH (15 Trevira vs. 15 no Trevira) using MUTARS^®^ silver-coated	FU: 26 mo (24–84) Complications: 1 PJI (Trevira, NS); compl. 20% Functional outcome: Enneking: 20/30; ROM comparable
***Henderson et al.*** [32]	2011	Literature review	2174 EP (348 PH, 16 TH) Trevira often used	FU up to 34 years Complications: failure in PH 17% (59/348); soft-tissue failure 4%; infection 6.3%; AL2.6%; structural failure 1.1%; Functional outcomes: not uniformly reported
***Lang et al.*** [21]	2021	Retrospective cohort	18 LT surv. (14 PH, 4 TH); 8 mesh Modular EP ± mesh (LARS ~ Trevira)	FU 18 years (6–29). Complications: mesh associated with higher soft-tissue complications and migration; functional outcomes: TESS 80.8, UCLA 7

Abbreviations: PH = proximal humerus; TH = total humerus; FU = follow-up; pts = patients; EP = endoprostheses; LT surv. = long-term survivors; AL = aseptic loosening; inf. = infection; PJI = periprosthetic joint infection; compl. = complications; amps = amputations; ROM = range of motion; MSTS = Musculoskeletal Tumor Society score; LARS: Ligament Advanced Reinforcement System. TESS = Toronto Extremity Salvage Score; UCLA = University of California Los Angeles activity score.

## Data Availability

Data sharing not applicable to this article as no datasets were generated or analyzed.
